# The Role of Vertical Transmission in the Control of Dengue Fever

**DOI:** 10.3390/ijerph16050803

**Published:** 2019-03-05

**Authors:** David Murillo, Anarina Murillo, Sunmi Lee

**Affiliations:** 1 Simon A. Levin Mathematical, Computational and Modeling Sciences Center, Arizona State University, Tempe, AZ 85287, USA; David.Murillo@asu.edu (D.M.); Anarina.Murillo@asu.edu (A.M.); 2 Department of Applied Mathematics, Kyung Hee University, Yongin 446-701, Korea; 3 Institute of Natural Sciences, Kyung Hee University, Yongin 446-701, Korea

**Keywords:** two-strain dengue model, vertical transmission, basic reproductive number, optimal control interventions

## Abstract

In this work, a two-strain dengue model with vertical transmission in the mosquito population is considered. Although vertical transmission is often ignored in models of dengue fever, we show that effective control of an outbreak of dengue can depend on whether or not the vertical transmission is a significant mode of disease transmission. We model the effect of a control strategy aimed at reducing human-mosquito transmissions in an optimal control framework. As the likelihood of vertical transmission increases, outbreaks become more difficult and expensive to control. However, even for low levels of vertical transmission, the additional, uncontrolled, transmission from infected mosquito to eggs may undercut the effectiveness of any control function. This is of particular importance in regions where existing control policies may be effective and the endemic strain does not exhibit vertical transmission. If a novel strain that does exhibit vertical transmission invades, then existing, formerly effective, control policies may no longer be sufficient. Therefore, public health officials should pay more attention to the role of vertical transmission for more effective interventions and policy.

## 1. Introduction

Dengue fever is one of the most important re-emerging vector-borne diseases. The primary vector, *Aedes aegypti* has endured several attempted eradication campaigns, but both the vector and the virus have revealed themselves to be extremely resilient to control measures. Due to rapid urbanization, global travel, and environmental change, public health officials in the world face enormous future challenges from emerging or re-emerging infectious diseases [1]. Over the next 20 years with the largest share of the international growth coming from the Asia and Latin America, regions where dengue is endemic, mass transportation is indeed an important factor in the long-range dispersal of dengue [2,3,4,5]. Dengue puts 40% of the global population at risk with 50 to 100 million infections per year [1]. Despite intensive vector control programs, many countries have experienced dengue re-emergence over the last few decades [1,6].

There are only two diseases that have been successfully eradicated: smallpox in 1979 and just recently rinderpest has been declared eradicated by the UN, due in large part to an effective vaccine and aggressive vaccination program [7]. Although vaccines exist for many other diseases, cost and even public perception can limit vaccine coverage, hamper the establishment of herd immunity, and preclude disease eradication [8,9,10]. In 2015, the first dengue vaccine was used in Mexico, however, the effectiveness of the dengue vaccine is still under investigation [11], thus the mitigation and prevention policies have focused on breeding site reduction (elimination of mosquito breeding sites) and spraying programs; that is, they have focused on controlling the vector [6]. The primary drivers of species extinction are habitat disturbance and direct elimination (harvesting, hunting, etc.) [12,13]. Botanical extracts have been pursued as an alternative means of vector control, but their effectiveness (as part of a dengue control strategy) has yet to be ascertained [14,15]. More recent work explored the impact of modern countermeasures such as the Sterile Insect Technique (SIT), the Release of Insects carrying Dominant Lethal genes (RIDL) and the release of Wolbachia-infected mosquitoes [16]. However, *Ae. aegypti* has demonstrated an affinity to the urban landscape and ability to thrive even in countries with strict control programs [3,17,18].

There are five distinct serotypes in dengue virus: DENV-1, DENV-2, DENV-3, DENV-4, and DENV-5 [1]. The disease symptoms range from asymptomatic, mild dengue fever (DF), to severe stages such as dengue hemorrhagic fever (DHF), and dengue shock syndrome (DSS) [19,20]. Dengue virus is mainly transmitted to humans through the bite of infected female mosquitoes of the Aedes species; this process is called horizontal transmission. Also, the mosquito becomes infected when it bites an infected human. However, there exists the possibility of vertical transmission of DENV from the infected female to her eggs (note that vertical transmission occurs in the mosquito population only not in the human population). Recent studies have shown clear evidence of vertical transmission of dengue in the mosquito population for *Ae. aegypti* and *Ae. albopictus* [21,22,23]. Moreover, other findings have explored that vertical transmission involving *Ae. aegypti* and *Ae. albopictus* species is feasible in captivity and in nature [24,25,26,27,28,29]. Vertical transmission provides a possible mechanism supporting virus dengue persistence in the absence of a recognized host and/or under unfavorable conditions for mosquito activity [30]. A literature review is performed on the presence of natural vertical transmission of DENV in *Ae. aegypti* and *Ae. albopictus* [31].

Mathematical modeling of vector-borne diseases has evolved from simpler models [32] to more complex models that include climate changes, socio-economic changes and urbanization [33]. Geographic heterogeneity and climate change are some of the key factors for recurrent vector-borne diseases in many tropical/subtropical countries [34]. Systematic reviews on mathematical and statistical models have been performed for the transmission dynamics of dengue [35,36]. Particularly, mathematical analyses of the role of vertical transmission were carried out in [22,37,38]. Their results confirmed the idea that vertical transmission can be an essential mechanism that favored the maintenance of the virus even with low human densities [37]. On the other hand, a substantial proportion of vertical transmission (when vertical transmission is over 20%) could enhance the persistence of the dynamics of dengue disease and otherwise, the role of vertical transmission was negligible [22].

Our work is motivated by the 2000–2001 dengue outbreak in Peru, where two strains of DENV-2 are co-circulating (American and Asian of the DENV-2 serotype), and particularly with vertical transmission in the Asian strain of DENV-2 [38]. An invading strain of dengue virus (DENV-2) from Asia rapidly circulated into Peru eventually displacing DENV-2 American. Some fields studies have demonstrated the percentage of natural vertical transmission of DENV from the female to her eggs by analyzing the presence of DENV in terms of the minimum infection rates (MIR) [39], suggesting that Aedes species display different susceptibilities to dengue virus infections. Laboratory experiments also have supported the hypothesis that higher infection rates exist when *Ae. aegypti* is exposed to the DENV-2 Asian strain in comparison to DENV-2 American [40]. The biological mechanisms behind the displacement of DENV-2 American by DENV-2 Asian at the population level was carried out in the previous work ([38] and references therein). Their results highlighted the importance of vertical transmission, observing that lower transmission rates of DENV-2 Asian are sufficient for displacing DENV-2 American in the presence of vertical transmission.

We suggest that vertical transmission, an often overlooked transmission pathway for dengue fever, may contribute to the difficulty of controlling the disease. While we have mentioned some of the numerous political and ecological reasons for the failure of previous eradication campaigns [9], this paper aims to elucidate some implications of vertical transmission on an attempt to control an outbreak of dengue fever. In the present work, we formulate an optimal control problem to identify optimal control strategies for a two-strain dengue model with vertical transmission in the mosquito population. Because vertical transmission is often considered to not be a major factor in dengue transmission, we model the effect of a control measure that does not directly impact vertical transmission. We then compare situations where vertical transmission is and is not a significant mode of dengue transmission. In the next section, we will develop the system with control and develop conditions for the existence of optimal control. Then we present some numerical results and finally we discuss the implications of optimal control in the two-strain model with vertical transmission.

## 2. A Two-Strain Dengue Model with Control

A two-strain dengue model was developed to assess the dynamics of two-strain competition, motivated by the context of the 2000–2001 dengue outbreak in Peru [38]. Previous to 2000 only DENV-1 and DENV-2 American genotypes had co-circulated in Peru with neither DHF nor DSS cases reported [41,42]. The absence of DHF and DSS in Peru prior to 2000, in the presence of co-circulating DENV-1 and DENV-2 American, had been explained, using the data of experiments carried out in laboratories. These studies identified partial cross-immunity conferred by DENV-1 against DENV-2 American but not conferred against the 2000–2001 invading DENV-2 Asian strain [41]. Currently, at least four dengue serotypes are found in Peru: DENV-1, DENV-2 (American and Asian), DENV-3, and DENV-4 serotypes. The displacement of DENV-2 American by the DENV-2 Asian has also been associated with the appearance of DHF in the Americas [43]. This shows biological evidence supporting the greater virulent strength of DENV-2 Asian.

Although vertical transmission has been mostly understudied in models of dengue, recent results [38] have demonstrated that vertical (transovarial) transmission has both primary and secondary effects in facilitating the invasion and persistence of novel strains of dengue. Dengue management policies exist virtually everywhere dengue fever is a major health concern, yet the fact that dengue outbreaks are increasing in severity and frequency suggests we need to better understand control strategies and how to evaluate them [44]. Among these features is vertical transmission which will be explored by considering a population that is impacted by two variants of the same serotype of dengue simultaneously: one that exhibits vertical transmission as a significant mode of disease transmission (DENV-2 Asian) and one that does not (DENV-2 American). In the present work, we extend the previous model [38] by incorporating a time-dependent control function.

**Figure 1:**
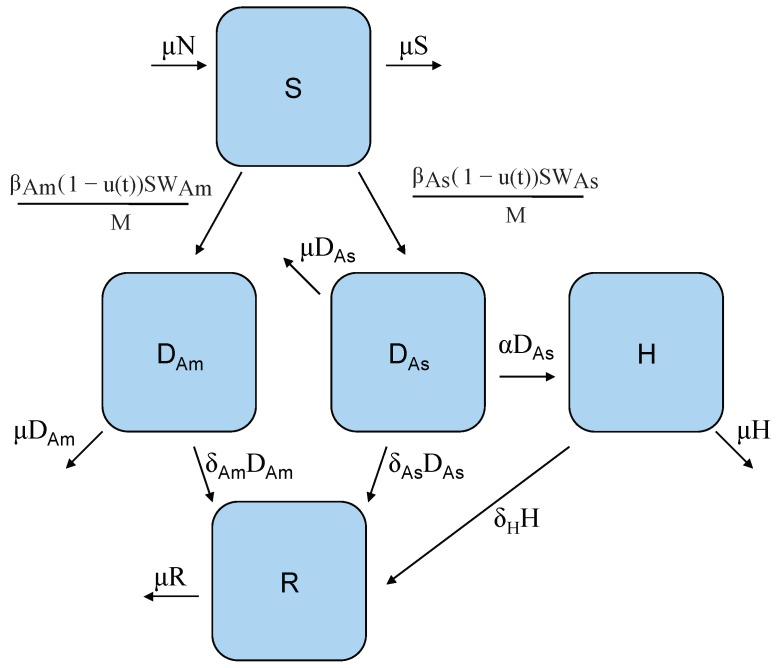
Host model flow diagram: *S* is the class of susceptible individuals who can become infectious with either DENV-2 American genotype, $D_{Am}$, or DENV-2 Asian genotype $D_{As}$ via infectious female mosquitoes *W* carrying the corresponding strain. In this model, only individuals infected with the Asian genotype can progress to DHF, *H*, and all infected individuals can recover, *R*. Note that the control function (1−u(t)) is modeled as the reduction efforts in the transmission rate from *S* either to $D_{Am}$, or $D_{As}$.

**Figure 2:**
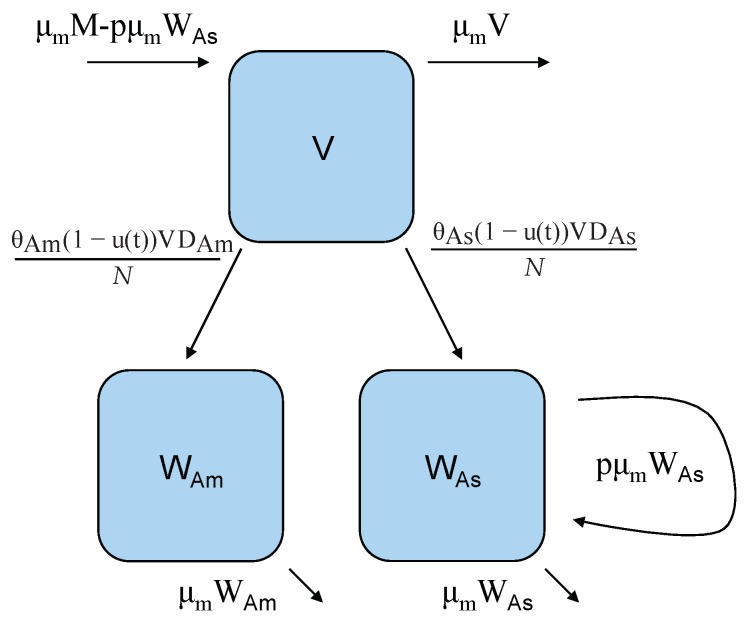
Vector model flow diagram: *V* is the class of susceptible female mosquitoes that can become infected with either DENV-2 American genotype $W_{Am}$ or DENV-2 Asian genotype $W_{As}$ via contact with an infectious human, *D* carrying the corresponding genotype. Vertical transmission only occurs in mosquitoes infected with genotype Asian. In this model, there is a constant birth rate, but a proportion, *p*, of those births by mosquitoes carrying genotype Asian, $W_{As}$, enter directly into the infectious class. Note that the control function (1−u(t)) is modeled as the reduction efforts in the transmission rate from *V* either to $W_{Am}$, or $W_{As}$.

We use a compartmental modeling framework where each compartment, shown in Figure 1 and Figure 2 by a letter within a box, denotes a class of individuals. Then the arrows represent the flows of individuals between different states [45]. Let *S* represent the number of susceptible hosts (humans). These individuals are antigenically naive to the particular strain of dengue being modeled but may have had previous exposures to other strains. $D_{Am}$ and $D_{As}$ are individuals infected with genotypes of dengue 2, DENV-2 Asian and DENV-2 American, respectively. *H* represents individuals who have developed DHF, *R* and is recovered individuals. *N* is the total human population size that is assumed constant since the change in population size is insignificant for a short time period. *V* is the class of susceptible vectors (female mosquitoes). $W_{Am}$ and $W_{As}$ are mosquitoes that carry strain DENV-2 American and DENV-2 Asian, respectively. *M* is the total vector population size, is assumed constant which is biologically reasonable within a short time scale as well. Then we can write the system of equations representing our model as:

(1) $$\begin{array}{ccc} \dot{S} & = & \mu N-\frac{\beta_{Am}\left(1-u\left(t\right)\right)SW_{Am}}{M}-\frac{\beta_{As}\left(1-u(t)\right)SW_{As}}{M}-\mu S\\ {\dot{D}}_{Am} & = & \frac{\beta_{Am}\left(1-u\left(t\right)\right)SW_{Am}}{M}-\left(\delta +\mu \right)D_{Am}\\ {\dot{D}}_{As} & = & \frac{\beta_{As}\left(1-u(t)\right)SW_{As}}{M}-\left(\delta +\alpha +\mu \right)D_{As}\\ \dot{H} & = & \alpha D_{As}-\left(\delta +\mu \right)H\\ \dot{R} & = & \delta D_{Am}+\delta D_{As}+\delta H-\mu R\\ \dot{V} & = & \mu_{m}M-p\mu_{m}W_{As}-\frac{\theta_{Am}\left(1-u\left(t\right)\right)VD_{Am}}{N}-\frac{\theta_{As}\left(1-u\left(t\right)\right)VD_{As}}{N}-\mu_{m}V\\ {\dot{W}}_{Am} & = & \frac{\theta_{Am}\left(1-u\left(t\right)\right)VD_{Am}}{N}-\mu_{m}W_{Am}\\ {\dot{W}}_{As} & = & \frac{\theta_{As}\left(1-u\left(t\right)\right)VD_{As}}{N}+p\mu_{m}W_{As}-\mu_{m}W_{As} \end{array}$$

Note that $\dot{N}=0$ and $\dot{M}=0$ when we add all the equations in the above system (1). In this work, a control function (*u*(*t*)) is modeled by preventive control efforts: preventive control efforts may involve the application of a pesticide (sprays), reduction of vector breeding grounds, mosquito repellents, or the results of education campaigns, which increase personal protection. It is assumed that these preventive interventions do not reduce the total vector population significantly, and the effect of these interventions implicitly translates in reductions of transmission between vectors and hosts per unit time.

Therefore, u(t) is the percentage reduction in infection due to the effect of control measures. Then $\beta_{i}\left(1-u\right)$ is the effective transmission force for strain *i*. Note we assume no a priori knowledge of what strain a particular individual has, thus the control measure is independent of the strain. Furthermore, since we are primarily interested in modeling the effect of a control measure, we assume that the reduction in effective contacts impacts mosquitoes equally well as humans. Thus, their effective force of infection is also reduced by u(t). We also assume that one strain, DENV-2 Asian, is more virulent, leading to cases of DHF and also exhibiting vertical transmission with some probability *p* times the basic fecundity function while the other strain, DENV-2 American, does not.

When the control function u(t)≡0, system (2) is said to be autonomous. The basic reproductive number of an epidemiological model generally determines whether or not the disease will die out or persist [45]. For the autonomous system, if we consider each strain independently, then the reproductive number for DENV-2 American is $R_{0}^{Am}=\sqrt{\frac{\beta_{Am}}{\left(\delta +\mu \right)}\frac{\theta_{Am}}{\mu_{m}}},$ and the reproductive number for DENV-2 Asian is $R_{0}^{As}=\frac{p}{2}+\sqrt{{\left(\frac{p}{2}\right)}^{2}+\frac{\beta_{As}}{\left(\delta +\alpha +\mu \right)}\frac{\theta_{As}}{\mu_{m}}}.$ Then the basic reproductive number is $R_{0}=max\left[R_{0}^{Am},R_{0}^{As}\right]$ (more detailed computations are found in [38]). The basic reproductive number is a central component of the model that can distinguish between different qualitative behavior in the autonomous system.

## 3. An Optimal Control Problem

A central component of the control problem is the optimization, in this case, minimization, of an objective function. We are interested in controlling an outbreak of dengue, thus we want to minimize the number of infected humans and the cost of implementing control efforts as well. However, we are also interested in preventing future outbreaks, thus we want to minimize the number of infected mosquitoes and individuals both during the course of our control measure and when our control policy has ended at time t=T. Then the corresponding objective function is:

(2) $$\begin{array}{ccc} J(u(t)) & = & \int_{0}^{T}\left(w_{1}\left(D_{Am}\left(t\right)+D_{As}\left(t\right)\right)+w_{2}\left(W_{Am}\left(t\right)+W_{As}\left(t\right)\right)+\frac{1}{2}w_{3}u^{2}\left(t\right)\right)dt\\ & & +w_{4}\left(D_{Am}\left(T\right)+D_{As}\left(T\right)\right)+w_{5}\left(W_{Am}\left(T\right)+W_{As}\left(T\right)\right) \end{array}$$

where $w_{1}$ is the weight constant for host infections, $w_{2}$ is the weight constant for vector infections. $\frac{1}{2}w_{3}u^{2}\left(t\right)$ is the cost of control with the weight constant $w_{3}$ and included as a quadratic term for the existence of optimal control due to the convexity of a control function in the objective function. Lastly, $w_{4}$ and $w_{5}$ are the weight constant for the payoff term (at the final time, t=T). If we let *X* be the vector of our state variables which is restricted to the positive orthant, $X\in R_{+}^{8}$, then $X^{*}$ is the optimal solution that corresponds to the optimal control function $u^{*}$ such that

$$J\left(u^{*}\right)=\min\left\{J\left(u\right)|u\in \Omega \right\},$$

where $\Omega =\{\left(u\left(t\right)\in L^{1}\;|\;0\leq u\left(t\right)\leq 1,t\in \left[0,T\right]\right\}.$ Then the Hamiltonian of our system is

(3) $$\begin{array}{ccc} \hat{H}\left(X,u\right) & = & w_{1}\left(D_{Am}\left(t\right)+D_{As}\left(t\right)\right)+w_{2}\left(W_{Am}\left(t\right)+W_{As}\left(t\right)\right)+\frac{1}{2}w_{3}u^{2}\left(t\right)\\ & & +\lambda_{1}\left(\mu N-\frac{\beta_{Am}\left(1-u\left(t\right)\right)SW_{Am}}{M}-\frac{\beta_{As}\left(1-u\left(t\right)\right)SW_{As}}{M}-\mu S\right)\\ & & +\lambda_{2}\left(\frac{\beta_{Am}\left(1-u\left(t\right)\right)SW_{Am}}{M}-\left(\delta +\mu \right)D_{Am}\right)\\ & & +\lambda_{3}\left(\frac{\beta_{As}\left(1-u\left(t\right)\right)SW_{As}}{M}-\left(\delta +\alpha +\mu \right)D_{As}\right)\\ & & +\lambda_{4}\left(\alpha D_{As}-\left(\delta +\mu \right)H\right)\\ & & +\lambda_{5}\left(\delta D_{Am}+\delta D_{As}+\delta H-\mu R\right)\\ & & +\lambda_{6}\left(\mu_{m}M-p\mu_{m}W_{As}-\frac{\theta_{Am}\left(1-u\left(t\right)\right)VD_{Am}}{N}-\frac{\theta_{As}\left(1-u\left(t\right)\right)VD_{As}}{N}-\mu_{m}V\right)\\ & & +\lambda_{7}\left(\frac{\theta_{Am}\left(1-u\left(t\right)\right)VD_{Am}}{N}-\mu_{m}W_{Am}\right) \end{array}$$

(4) $$\begin{array}{c} +\lambda_{8}\left(\frac{\theta_{As}\left(1-u\left(t\right)\right)VD_{As}}{N}+p\mu_{m}W_{As}-\mu_{m}W_{As}\right), \end{array}$$

where $\lambda_{i}$ are the co-state or adjoint variables [46]. Then, by Pontryagin’s Maximum Principle [47], our optimal solution can be found by simultaneously solving the adjoint system:

$$\begin{array}{ccc} \frac{d\lambda_{1}\left(t\right)}{dt} & =-\frac{\partial \hat{H}}{\partial S}= & \left(\lambda_{1}-\lambda_{2}\right)\beta_{Am}\left(1-u\right)\frac{W_{Am}}{M}+\left(\lambda_{1}-\lambda_{3}\right)\beta_{As}\left(1-u\right)\frac{W_{As}}{M}+\lambda_{1}\mu \\ \frac{d\lambda_{2}\left(t\right)}{dt} & =-\frac{\partial \hat{H}}{\partial D_{Am}}= & \left(\lambda_{6}-\lambda_{7}\right)\theta_{Am}\left(1-u\right)\frac{V}{N}+\left(\lambda_{2}-\lambda_{5}\right)\delta +\lambda_{2}\mu -w_{1}\\ \frac{d\lambda_{3}\left(t\right)}{dt} & =-\frac{\partial \hat{H}}{\partial D_{As}}= & \left(\lambda_{6}-\lambda_{8}\right)\theta_{As}\left(1-u\right)\frac{V}{N}+\left(\lambda_{3}-\lambda_{5}\right)\delta +\left(\lambda_{3}-\lambda_{4}\right)\alpha +\lambda_{3}\mu -w_{1}\\ \frac{d\lambda_{4}\left(t\right)}{dt} & =-\frac{\partial \hat{H}}{\partial H}= & \left(\lambda_{4}-\lambda_{5}\right)\delta +\lambda_{4}\mu \\ \frac{d\lambda_{5}\left(t\right)}{dt} & =-\frac{\partial \hat{H}}{\partial R}= & \lambda_{5}\mu \\ \frac{d\lambda_{6}\left(t\right)}{dt} & =-\frac{\partial \hat{H}}{\partial V}= & \left(\lambda_{6}-\lambda_{7}\right)\theta_{Am}\left(1-u\right)\frac{D_{Am}}{N}+\left(\lambda_{6}-\lambda_{8}\right)\theta_{As}\left(1-u\right)\frac{D_{As}}{N}+\lambda_{6}\mu_{m}\\ \frac{d\lambda_{7}\left(t\right)}{dt} & =-\frac{\partial \hat{H}}{\partial W_{Am}}= & \left(\lambda_{1}-\lambda_{2}\right)\beta_{Am}\left(1-u\right)\frac{S}{N}+\lambda_{7}\mu_{m}-w_{2}\\ \frac{d\lambda_{8}\left(t\right)}{dt} & =-\frac{\partial \hat{H}}{\partial W_{As}}= & \left(\lambda_{1}-\lambda_{3}\right)\beta_{As}\left(1-u\right)\frac{S}{N}+\left(\lambda_{6}-\lambda_{8}\right)p\mu_{m}+\lambda_{8}\mu_{m}-w_{2}, \end{array}$$

with the transversality conditions at t=T

$$\begin{array}{c} \lambda_{1}=\lambda_{4}=\lambda_{5}=\lambda_{6}=0\\ \lambda_{2}=\lambda_{3}=w1\\ \lambda_{7}=\lambda_{8}=w2, \end{array}$$

and the optimality condition

(5) $$\begin{array}{ccc} \frac{\partial \hat{H}}{\partial u} & = & w_{3}u+\left(\lambda_{1}-\lambda_{2}\right)\beta_{Am}S\frac{W_{Am}}{M}+\left(\lambda_{1}-\lambda_{3}\right)\beta_{As}S\frac{W_{As}}{M}\\ & & +\left(\lambda_{6}-\lambda_{7}\right)\theta_{Am}V\frac{D_{Am}}{N}+\left(\lambda_{6}-\lambda_{8}\right)\theta_{As}V\frac{D_{As}}{N}, \end{array}$$

where $\frac{\partial \hat{H}}{\partial u}=0$ at $u=u^{*}.$ We can solve this for the optimal control function $u^{*}$ with the constraint that *u* must be between 0 and 1 to get

$$\begin{array}{ccc} u^{*} & = & \min\left\{\max\left\{0,\left(\lambda_{2}-\lambda_{1}\right)\beta_{Am}S\frac{W_{Am}}{w_{3}M}+\left(\lambda_{3}-\lambda_{1}\right)\beta_{As}S\frac{W_{As}}{w_{3}M}\right.\right.\\ & & \left.\left.+\left(\lambda_{7}-\lambda_{6}\right)\theta_{Am}V\frac{D_{Am}}{w_{3}N}+\left(\lambda_{8}-\lambda_{6}\right)\theta_{As}V\frac{D_{As}}{w_{3}N}\right\},1\right\}. \end{array}$$

This type of optimal control formulation has several applications in mathematical biology [46,48,49,50,51]. Although proof of the existence of optimal control is left to the Appendix A, the solution to our control problem will be a piecewise smooth control function. For the purposes of this article, what is important is the qualitative shape of this control function. Because it is unclear what the costs of these control policies are relative to the effective reduction in transmission, more insight may be gleaned by examining the qualitative features of the control function as the relative costs are changed.

## 4. Numerical Results

Each numerical solution is performed over a period of three years to give account for transient dynamics. In reality, a control policy would also be evaluated over short, medium and long term time periods, and three years seemed sufficient for our numerical results. The default parameters for all simulations are listed in Table 1 unless otherwise indicated.

### 4.1. The Impact of the Relative Cost on the Controlled Dengue Dynamics

**Figure 3:**
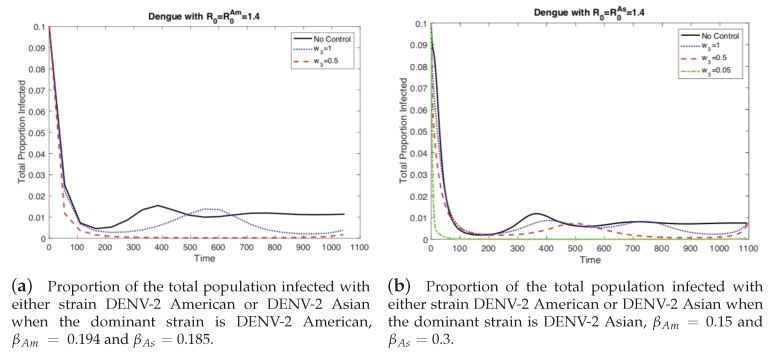
As the relative cost of the control function, *w*_3_, is reduced, the proportion of infected people decreases. However when the outbreak is dominated by the strain without vertical transmission, (**a**) then the outbreak can be controlled more easily than when the outbreak is dominated by the strain with vertical transmission, (**b**) In the latter case, the cost of control must be reduced even further to effectively control the outbreak.

**Figure 4:**
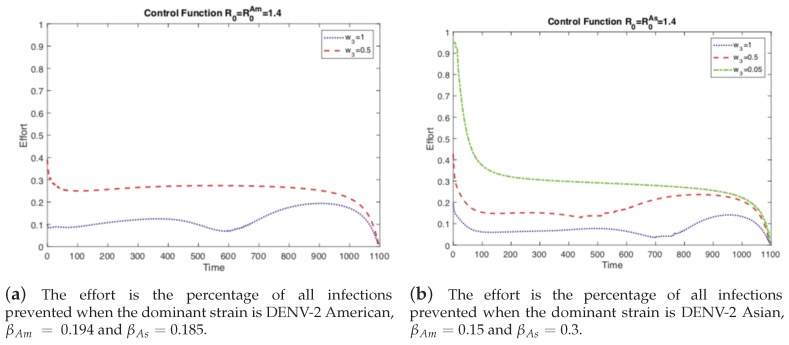
As the relative cost of the control function decreases, it is used more frequently and is able to control the outbreak. If the relative cost is expensive, then it is used sparingly and in response to outbreaks. Notice the peaks occur right after an increase in the prevalence of dengue in the corresponding panel of Figure 3.

First, we investigate the impact of different values of the relative cost of control ($w_{3}$) on the controlled dengue dynamics. The weight constant can be considered as the relative cost of control implementation, and a larger value represents a relatively higher cost. Figure 3 and Figure 4 illustrate the impact of control weight constants under several values of $w_{3}=1,0.5,0.05$. As mentioned in the previous section, there are two cases of the basic reproduction number: either one of the two strains is dominant ($R_{0}=max\left[R_{0}^{Am},R_{0}^{As}\right]$). Overall, the impact is straightforward; for higher costs, the control decreases, which leads to larger outbreaks.

If the cost of the control function is comparable, on the same order of magnitude ($w_{3}=1$), to the costs incurred from the disease, then there is no incentive to invest heavily on control. We see this in Figure 3 where not much effort is spent on the control function. However, if the control becomes less expensive, or analogously the costs from disease become more expensive, then it is worthwhile to invest in eliminating the disease and preventing an outbreak. Note that with sufficient effort the control function can mitigate the current outbreak and prevent future ones (the damped oscillations predicted in the autonomous model) as seen in Figure 3. This is the case where the basic reproductive number, $R_{0},$ is greater than one (1.4), otherwise there would be no outbreak and control would be moot.

However, the left panels in Figure 3 and Figure 4 are when $R_{0}^{As}<R_{0}^{Am},$ i.e., the strain without vertical transmission is the dominant strain during an outbreak. If we keep the same basic reproductive number but instead chose the outbreak to be dominated by the strain with vertical transmission, $R_{0}^{As}>R_{0}^{Am},$ then we get the scenarios depicted in the right panels of Figure 3 and Figure 4. Here we see that when the cost of control is comparable to the cost of the disease, we get the same results as before. When the cost of control is too high, we cannot completely control the outbreak and we must respond to rises in prevalence, Figure 4. However, at the same level of relative costs where the outbreak was controlled before, here we are unable to fully control the outbreak. The total number of cases is larger and there is a small secondary outbreak. In order to fully control the outbreak, we have to reduce the relative costs even further than in the previous case. Vertical transmission (*p* the proportion of eggs hatched infected with dengue) made the outbreak more difficult to control because the control function did not prevent the development of newly infected mosquitoes from infected eggs.

### 4.2. The Impact of Vertical Transmission on the Controlled Dengue Dynamics

**Figure 5:**
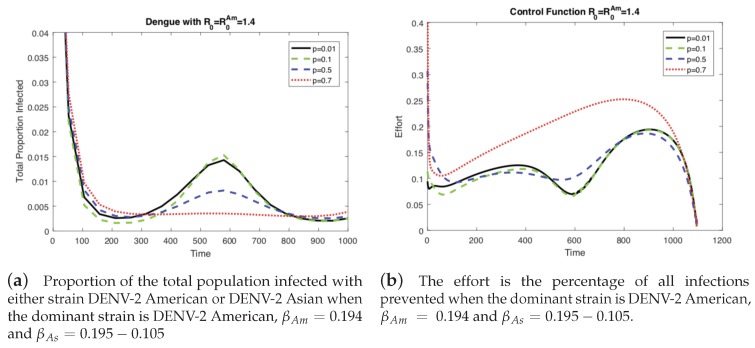
When the dominant strain is DENV-2 American, as the level of vertical transmission increases, the level of optimal control increases (**b**). Therefore, it is easier to control the outbreak (**a**).

**Figure 6:**
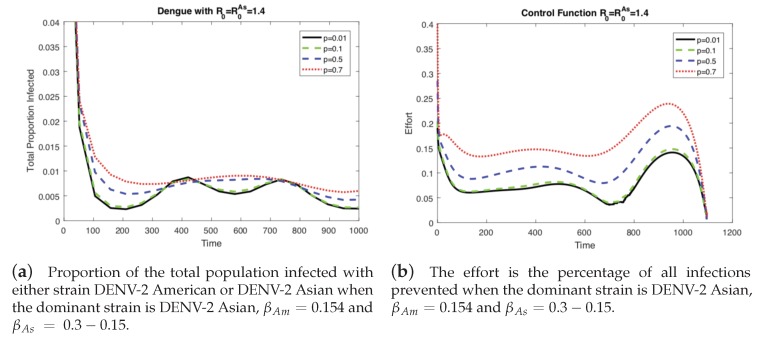
When the dominant strain is DENV-2 Asian, as the level of vertical transmission increases, the level of optimal control increases (**b**). However, it is harder to control the outbreak (**a**).

In the previous results (Figure 3 and Figure 4), the level of vertical transmission was relatively low (*p* = 0.0103). Now, we investigate the impact of vertical transmission on the controlled dengue dynamics (four different values of p=0.01,0.1,0.5,0.7 are used). Figure 5 and Figure 6 display the total proportion of infected and optimal controls under four different values of *p* using the same value of $w_{3}=1$ and the same value of $R_{0}=1.4$. Note that in order to keep the same value of $R_{0}$, $\beta_{1}$ and $\beta_{2}$ are varied as well. Again, we present two cases of the basic reproduction number: either one of the two strains is dominant ($R_{0}=max\left[R_{0}^{Am},R_{0}^{As}\right]$), which are displayed in Figure 5 and Figure 6.

In Figure 5 (when the American strain is dominant), the impact is straightforward; for a higher vertical transmission rate, the control increases (b), which leads to smaller outbreaks (a). Interestingly, Figure 6 (when the Asian strain is dominant) shows a counterintuitive effect of *p*; for a higher vertical transmission rate, even though the control increases (b), it becomes harder to control the outbreak (a). This confirms that, in particular, when the dominant strain is Asian, a higher vertical transmission rate increases the difficulty of controlling the outbreak under the same level of $R_{0}=1.4$.

### 4.3. The Impact of Vertical Transmission on the Objective Function

**Figure 7:**
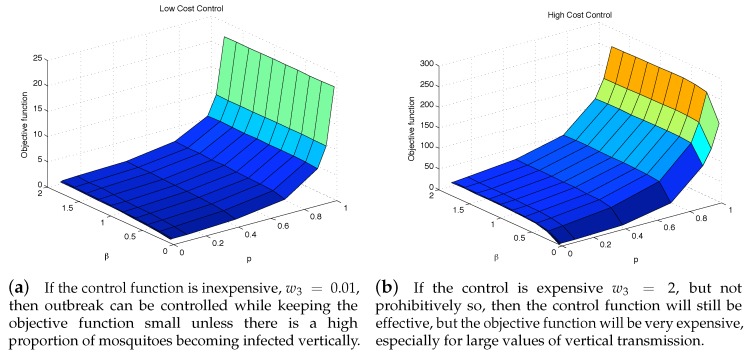
Even with an “effective” control program, a high vertical transmission rate can render the health policy moot regardless of the cost of additional control is low (**a**), or high (**b**).

**Figure 8:**
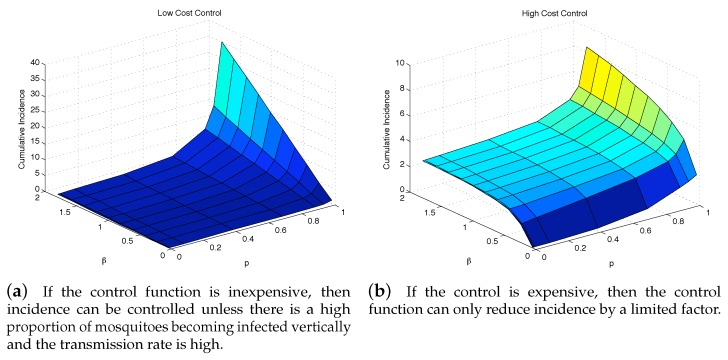
The outbreak can be well controlled except when vertical transmission is extremely high. Although this situation is unrealistic, it highlights the importance of a control strategy, whether highly cost-efficient, left, or otherwise to take into consideration all possible transmission pathways.

To further see the impact of vertical transmission, we measured the total value of the objective function and the cumulative incidence as functions of *p* and $\beta_{2}$. Figure 7 and Figure 8 illustrate the results under two weight constants (low cost using $w_{3}=0.01$ and high cost using $w_{3}=2$). If the relative cost of control is higher, then the total costs are proportionally higher as well. Regardless of the costs of control, having a large force of vertical transmission makes an outbreak extremely expensive to control, Figure 7b. This is due to the fact that the control policy cannot directly stop the generation of infected mosquitoes via vertical transmission, and thus are penalized by the number of new infections those mosquitoes cause, Figure 8b. As seen in both Figure 7 and Figure 8, under the parameter values used here, the impact of *p* on the objective function value and the cumulative incidence is more significant than the impact of $\beta_{2}$. In the low-cost case, the outbreak is manageable except *p* is very high (higher than 80%). On the other hand, the high-cost case, the outbreak is manageable only when p∈[0,30]% and $\beta_{2}\in \left[0,0.5\right]$.

**Figure 9:**
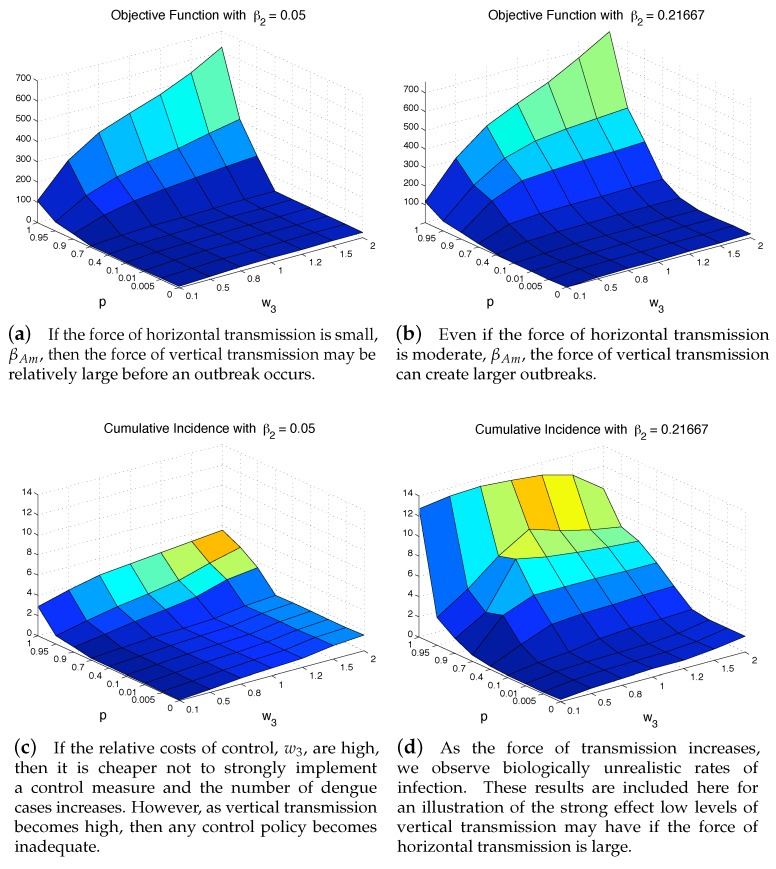
Regardless of whether horizontal transmission is low, left panels, or moderate, right panels, a high level of vertical transmission can create extremely large, and costly outbreaks, top panels. If the relative costs of controlling the outbreak are low, *w*_3_, then the epidemic can still be controlled, bottom panels. However, if the cost is high, then the outbreak will be extremely expensive and impossible to control.

For a fixed force of horizontal transmission, $\beta_{2}$, we can see how the total costs of control and the severity of an outbreak vary directly with changes in the force of vertical transmission, *p*, and relative cost of control, $w_{3}$. Figure 9 displays the results under two horizontal transmission rates (low using $\beta_{2}=0.05$ and high using $\beta_{2}=0.21667$). As the horizontal transmission increases ($\beta_{2}$), we notice that larger outbreaks occur for smaller values of vertical transmission, Figure 9d. Large values of vertical transmission can cause larger outbreaks with associated larger costs Figure 9b. For the lower horizontal transmission case, the outbreak is manageable except *p* is very high (over 70%) while the outbreak is manageable only when p∈[0,10]% for the higher horizontal transmission case.

## 5. Discussion

We developed an optimal control framework to identify optimal control strategies for a two-strain dengue model with vertical transmission in the mosquito population. Our model is motivated by the 2000–2001 dengue outbreak in Peru, where two strains of DENV-2 are co-circulating, and particularly with vertical transmission in the Asian strain of DENV-2. We evaluate the role of vertical transmission in the controlled dengue dynamics. Our results indicate that the controlled dengue dynamics are strongly dependent on the following three key factors: *p*, $\beta_{2}$, and $w_{3}$. Overall, controlling the outbreaks is more difficult as vertical transmission (*p*), $\beta_{2}$, and the relative cost increase.

Under the moderate level of $R_{0}=1.4$, the outbreak can be well controlled (when vertical transmission also is moderate). Especially, for the case of unlimited resources available (the relative cost is inexpensive $w_{3}=0.01$), controlling the outbreak is sufficiently effective even when *p* is high (p∈[0,70]%). As the relative cost becomes more expensive, controlling the outbreak is effective only in the smaller range of $\beta_{2}$ and *p*. If the cost is high ($w_{3}=2$), then the outbreak will be extremely expensive and impossible to control ($\beta_{2}>0.05$ and p>10%). Regardless of the relative cost of the control, outbreaks are extremely hard to manage when both horizontal and vertical transmission becomes higher. Finally, it is impossible at all as all of them increase ($w_{3}>1$, $\beta_{2}>0.2$ and p>10%). Although this situation is unrealistic, it highlights the importance of a control strategy, whether highly cost efficient or otherwise to take into consideration all possible transmission pathways.

Moreover, our findings highlight the importance of vertical transmission in the two-strain dengue dynamics. The two-strain model considers the competing dynamics of these two DENV-2 strains (the resident or the American type and the invasive more virulent Asian strain). The three critical factors mentioned above (*p*, $\beta_{2}$, and $w_{3}$) play a more significant role when the outbreak is dominated by the invasive DENV-2 Asian strain (i.e., $R_{0}^{As}>R_{0}^{Am}$). Since data from the 2000–2001 outbreak in Peru showed that DENV-2 Asian had displaced DENV-2 American [42], more careful prevention plans should be implemented when DENV-2 Asian strain is dominant.

As the relative cost of the control function is reduced, the proportion of infected people decreases. However, when the outbreak is dominated by the strain without vertical transmission, then the outbreak can be controlled more easily than when the outbreak is dominated by the strain with vertical transmission. In this case, the role of vertical transmission rate becomes negligible. Therefore, the effectiveness of control is strongly sensitive to various factors including the relative cost, dominant strains, the level of horizontal transmission, and vertical transmission. There are also similar results, observing that the role of vertical transmission is sensitive to other various factors [22].

Diseases have been and continue to be a major public health challenge, with outbreaks of infectious diseases capable of causing tremendous loss of life in relatively short periods. There are various strategies to controlling an epidemic (including vaccination, isolation, and social distancing) that have been used to study disease prevention/mitigation in various contexts (see [8,9,45,46,49] and references therein). Note that the current model could incorporate different control measures such as vaccination, treatments, chemical insecticide for adult mosquitoes or destruction of breeding sites (i.e., killing immature and aquatic stages). Some of these countermeasures have been implemented and compared in an optimal control framework [54,55,56]. Instead, our control is modeled as the effect of such preventive countermeasures mentioned above. We assumed that these preventive interventions do not reduce the total vector population significantly, and the effect of these interventions implicitly translates in reductions of transmission between vectors and hosts.

This implicit approach of control is employed, so the role of vertical transmission is made transparent as possible. However, the current study with such a simple assumption has limitations. For instance, the application of a pesticide (sprays), or reduction of vector breeding grounds will change the vector population size and the life-span of vector as well. Similarly, other control methods (mosquito repellents, or the results of education campaigns) will have different impacts on dengue transmission dynamics. It requires to develop relevant mathematical models, then to drive resulting optimality systems. Therefore, further extensive simulations and analyses should be carried out in future work. Furthermore, as the epidemiological and morbidity burden associated with dengue increase substantially, it becomes more critical to measure estimates of health and economic costs of the disease [5,57,58]. Extensive cost-effectiveness analyses based on real dengue burden should be carried out in future research.

## 6. Conclusions

We have modified the previous model proposed in [38] by incorporating a time-dependent control function. Our model is motivated by the 2000–2001 dengue outbreak in Peru, where an invading strain of dengue virus (DENV-2) from Asia rapidly circulated into Peru eventually displacing DENV-2 American. As the likelihood of vertical transmission increases, outbreaks become more difficult and expensive to control. This is of particular importance in regions where existing control policies may be effective, and the endemic strain does not exhibit vertical transmission.

This paper illuminates some of the implications of a control strategy that ignores the role of vertical transmission. If horizontal transmission is the dominant mode of transmission, and the moderate level of the basic reproductive number combined with the inexpensive cost of control, then the impact of vertical transmission may be negligible, and hence, the dengue outbreak is manageable. However, if any of those conditions are not met, vertical transmission may render a perfectly adequate control policy useless.

There is some evidence that genetic changes in either the vector or the virus may facilitate vertical transmission [24,26,28,59]. The unbeknownst proliferation of these genetic mutants can establish an alternative pathway of dengue transmission leading to unexpected outbreaks and perplexing regulators using policies that should be effective. Since the force of vertical transmission can increase both the costs associated with controlling the vector and the burden of dengue cases, public health officials should pay more attention to the role of vertical transmission for more effective interventions and policy.

## Figures and Tables

**Table 1:** Default Parameter Values: Biological parameters may vary across geographic and temporal scales, however, most of the values are taken from related literature or estimated to achieve the desired reproductive number.

Parameter	Default Value	Units	Source
*M*	1	per day	[52]
*N*	1	per day	[52]
α	0.113	per day	[52]
$\mu_{m}$	0.0958	per day	[53]
*p*	0–1	proportion	[27]
μ	0.00038	per day	estimated
$\theta_{As}$	0.28	per day	estimated
θAm	0.28	per day	estimated
$\beta_{As}$	0.01–0.2	per day	estimated
$\beta_{Am}$	0.01–0.2	per day	estimated
δ	0.2	per day	estimated

## Appendix A. Existence of Optimal Control

The optimal control exists under very general constraints. First note that solutions are bounded in the positive orthant: the derivative is non-negative on the zero boundary and $\frac{dN}{dt}=0$, $\frac{dM}{dt}=0$ where *N* is the sum of the host variables and *M* is the sum of the vector variables. The optimal control exists if the following conditions are satisfied [60]:

1. The set of controls and corresponding state variables is non-empty.
2. The control set, Ω, is convex and closed.
3. The right hand side of system (2) is bounded by a linear function in the state and control.
4. The integrand of the objective functional is convex and bounded below by $c_{1}\left(\right|u_{1}{|}^{2}+|u_{2}{{|}^{2})}^{\frac{\beta }{2}}-c_{2}$ and the Lipschitz condition is satisfied.
5. The payoff function is continuous.

**Proof**

1. If we consider the vector of state variables $x\;=\;{\left[S,\;D_{Am},\;D_{As},\;H,\;R,\;V,\;W_{Am},\;W_{As}\right]}^{T}$, then we can write our system of equations as
  $$\dot{x}\;=\;f\left(x,u\right).$$
  Since we know our state variables are bounded in the positive orthant, the particular form of our system of equations dictates that f(x,u) is bounded. Thus there exists a unique solution to our system given suitable initial conditions.
2. By the construction of Ω, this condition is clearly met.
3. The total population for both the host and vector systems is constant, thus all solutions are bounded. The control function is also bounded, thus the right-hand side can be bounded by a linear function in the state and control.
4. The integrand is linear in the state variable and quadratic in the control function, and thus clearly convex. Furthermore, the Lipschitz is condition is clearly satisfied as the integrand is bounded below since both the state and control are non-negative.
5. The payoff function is clearly continuous by construction.
